# Parvimonas micra: A Rare Cause of Pleural Empyema With COVID-19 Co-infection

**DOI:** 10.7759/cureus.51998

**Published:** 2024-01-10

**Authors:** Shamon Gumbs, Ifeoma Kwentoh, Eric Atiku, Winnie Gikunda, Ali Safavi

**Affiliations:** 1 Department of Surgery, Columbia University College of Physicians and Surgeons, Harlem Hospital Center, New York, USA; 2 Department of Medicine, Columbia University College of Physicians and Surgeons, Harlem Hospital Center, New York, USA

**Keywords:** thoracotomy, community acquired pneumonia, parvimonas micra, covid-19, empyema

## Abstract

*Parvimonas micra*, an oral anaerobe and a known gastrointestinal microbiota, has also been found to be enriched in mucosal tissues of the colon. Our patient presented with chest pain, productive cough, and hypoxia. He was diagnosed with COVID-19 pneumonia with a suspected superimposed bacterial infection. After the initiation of treatment, the patient developed a right hydropneumothorax/loculated pleural effusion on X-ray. Bedside drainage was done, and cross-sectional imaging showed findings of pleural empyema. Cultures obtained after bedside drainage grew *P. micra. *The patient underwent right posterolateral open thoracotomy, total lung decortication, wedge resection, pneumonolysis, and mechanical pleurodesis. Antimicrobial therapy was adjusted based on culture sensitivities and infectious disease evaluation. Adequate drainage and source control were achieved, COVID-19 infection was resolved, and the patient was discharged on oral antibiotics. This case report highlights a rare and interesting case of pleural empyema caused by a superimposed bacterial infection with *P. micra* in a patient with COVID-19 pneumonia.

## Introduction

*Parvimonas micra* is a gram-positive coccus, an obligate anaerobe that resides dominantly in the human oral cavity, which rarely causes pneumonia or empyema, and is often challenging to identify in laboratory testing [1,2]. Our patient presented with COVID-19 pneumonia and suspected bacterial superinfection which medical management was attempted but failed. The patient subsequently had thoracotomy with decortication, with appropriate source control and clinical improvement on tailored antimicrobial therapy. Based on our literature review, there were no cases of pleural empyema and co-infection with the COVID-19 virus that have been reported in the literature. This case report highlights the pathogenicity of *P. micra* and its potential association with COVID-19 co-infection. The study emphasizes the importance of early diagnosis and appropriate management of this rare condition to improve patient outcomes and reduce morbidity and mortality.

## Case presentation

A 50-year-old Hispanic male who resided in a shelter had a past medical history of active cigarette smoking (17.5 pack year), poly-substance use disorder (cocaine and heroin), and hyperlipidemia and presented to the emergency room with chest pain and productive cough for two weeks before presentation. There were no recent sick contacts, only a recent trip to Florida. In the emergency room, the patient reported having worsened chest pain with cough and occasional hypoxia. Physical exam was notable for mild tachypnea, aeration decrease on the right, rhonchi, and rales present bilaterally.

He was hemodynamically stable with SpO2 >95%. WBC elevated, neutrophil % elevated, lactate within normal range, elevated pro-inflammatory markers: pro-calcitonin, ferritin, C-reactive protein, lactate dehydrogenase (LDH), and D-dimer; with elevated pro-brain natriuretic peptide (pro-BNP) (Table 1).

**Table 1:** Table showing the laboratory values and trends throughout hospitalization. HD - Hospital day, WBC - White blood cell, Pro-BNP - Pro-brain natriuretic peptide, LDH - Lactate dehydrogenase

Lab test	Reference	HD 1	HD 3	HD 5	HD 12
WBC (x10^3^/mcL)	4.80-10.80	29.25	37.87	48.63	17.22
Neutrophil %	44.0-70.0	86.5	85.6	85.1	74.6
C-reactive protein (mg/L)	0.0-5.0	327.9	___	___	___
Procalcitonin (ng/mL)	0.02-0.08	1.28	___	___	0.21
Pro-BNP (pg/mL)	<=125	392	___	___	___
Ferritin (ng/mL)	30-400	1223	___	___	___
LDH (U/L)	135-225	280	___	458	___
D-dimer (ng/ml DDU)	<=250	791	888	___	___
Lactate (mmol/L)	0.6-1.4	1.6	2.7	___	1.5

**Figure 1:**
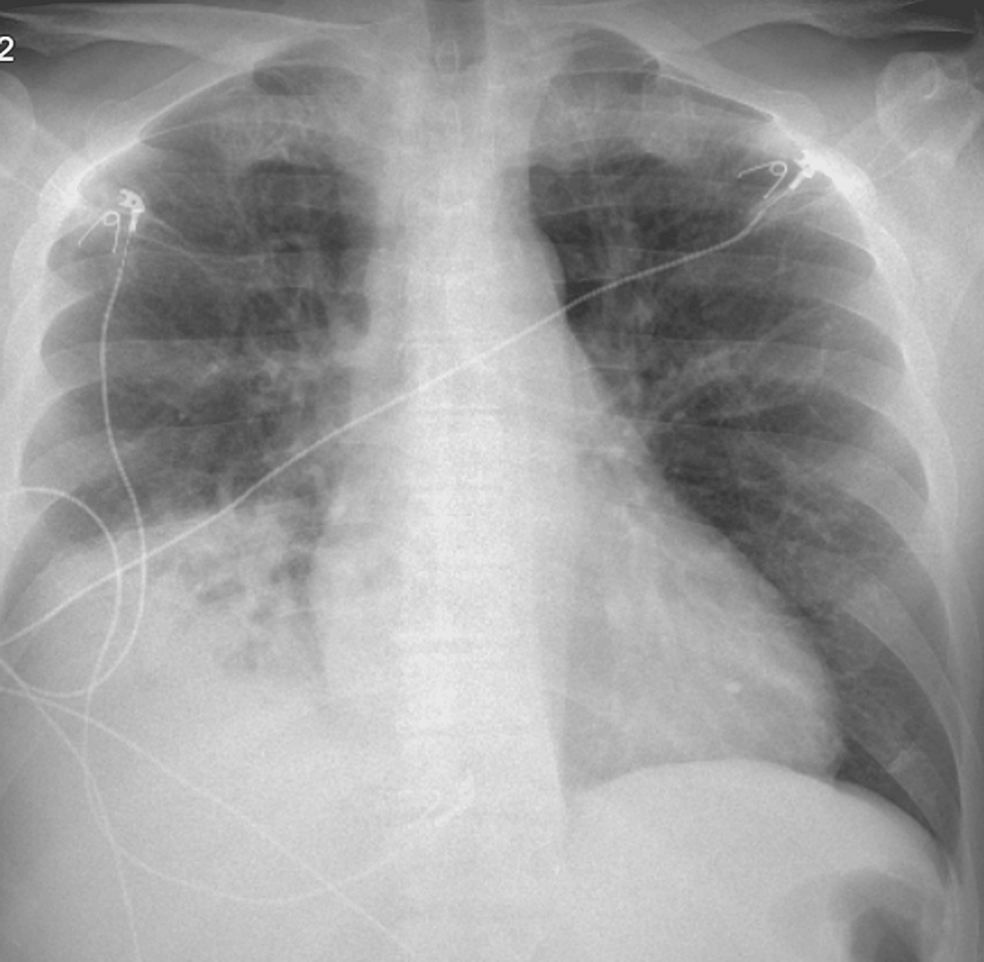
AP chest X-ray showing hazy opacification of the right lower lung field; areas of lucency in the medial margin of the consolidation. AP: Anteroposterior

Chest X-ray showed hazy opacification of the right lower lung field; areas of lucency in the medial margin of the consolidation may correspond to the aerated lung; however, necrosis can have a similar appearance (Figure 1). ECG showed normal sinus rhythm.

**Figure 2:**
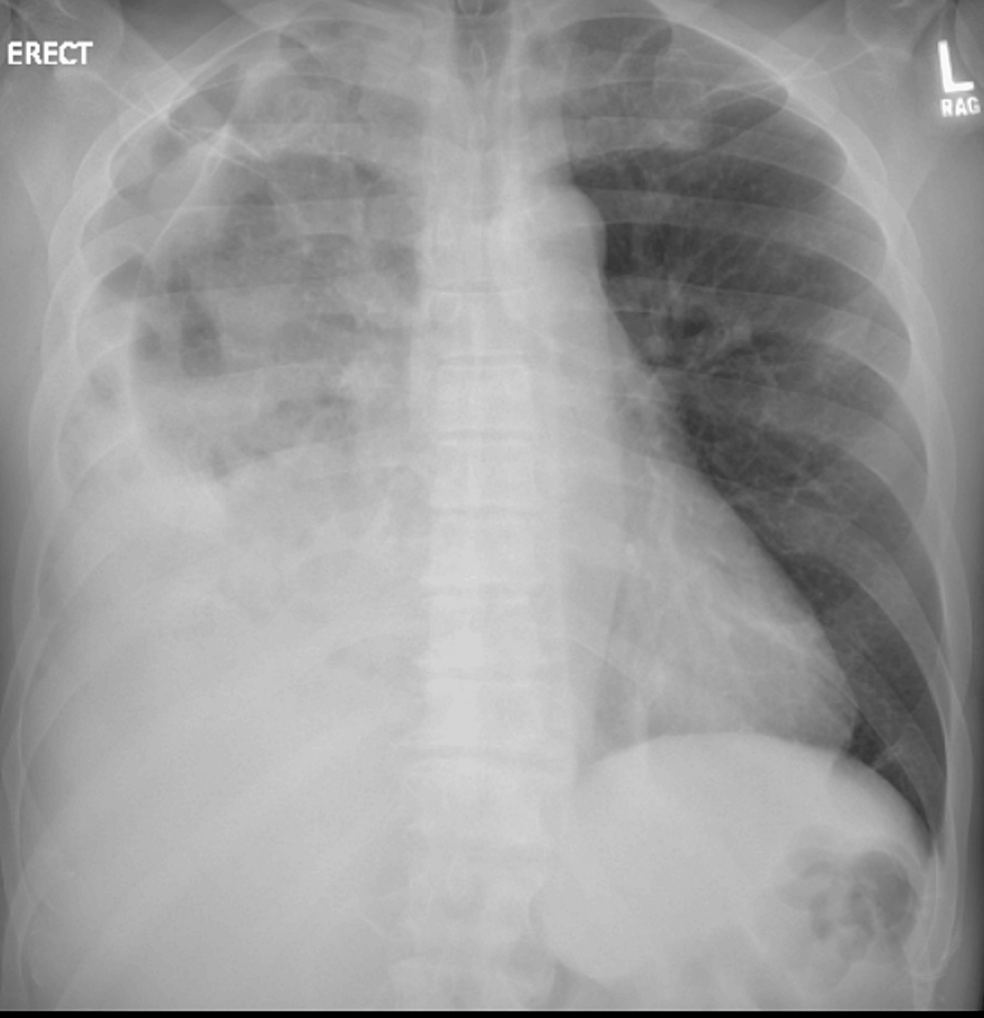
AP chest X-ray showing right moderate-size pneumothorax and a loculated pleural effusion. AP: Anteroposterior

The patient was found to have COVID-19 pneumonia with a suspected superimposed bacterial infection. This is because the presentation and imaging findings suggested a possible community-acquired pneumonia. The patient was admitted to the medical floor in isolation. He was initiated on COVID-19/community-acquired pneumonia treatment with remdesivir, ceftriaxone, and azithromycin. Critical care was consulted on hospital day (HD) 2 due to the patient having right-sided chest pain tachypnea/respiratory distress. The patient was started on non-invasive positive pressure ventilation. Thoracic surgery was consulted on HD 5 due to the development of a right moderate-size pneumothorax and a loculated pleural effusion seen on the chest X-ray (Figure 2). He remained hemodynamically stable, afebrile, and had an up-trending (Table 1), also on steroids.

A 32 F chest tube was placed in the right thoracic cavity and had 280 cc of cloudy-yellow fluid. Body fluid culture later grew *P. micra*. A chest CT without IV contrast was obtained and showed moderate right hydropneumothorax with a frothy appearance of fluid components and consolidation of the right middle and lower lobes with scattered air foci, possibly representing cavitation (Video 1).

**Video 1:** Axial view CT chest without contrast showing moderate right hydropneumothorax with a frothy appearance of fluid components and consolidation of the right middle and lower lobes with scattered air foci, possibly representing cavitation.

**Figure 3:**
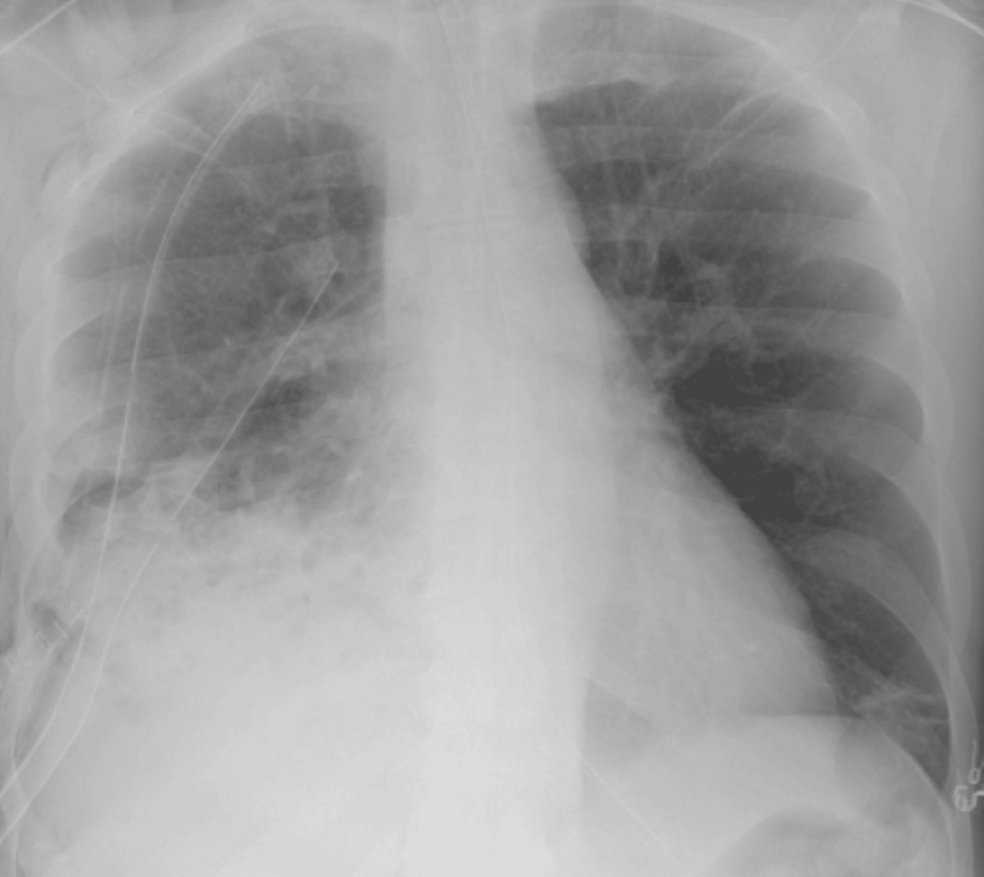
Post-operative AP chest X-ray showing improvement in the right-sided opacities and blunting of the right costophrenic angle. AP: Anteroposterior

A tissue plasminogen activator (tPA) was instilled via chest tubes without much improvement. The decision was made to proceed with surgery. The patient underwent right postero-lateral open thoracotomy, total lung decortication, wedge resection of the right lower and middle lobe, pneumonolysis, and mechanical pleurodesis on HD 7. Intraoperatively, he was found to have multiple loculated abscesses, dense adhesions, and fibrosis. Pleural fluid was neutrophilic exudate, and cultures grew *P. micra*. Post-operative chest X-ray showed improvement in the right-sided opacities and blunting of the right costophrenic angle (Figure 3).

Antibiotics were changed to levofloxacin, vancomycin, and Zosyn on post-op day (POD) 0. Infectious disease was consulted and agreed with management, but recommended a three- to six-week course of therapy. Surgical swab culture grew rare *P. micra*.

**Figure 4:**
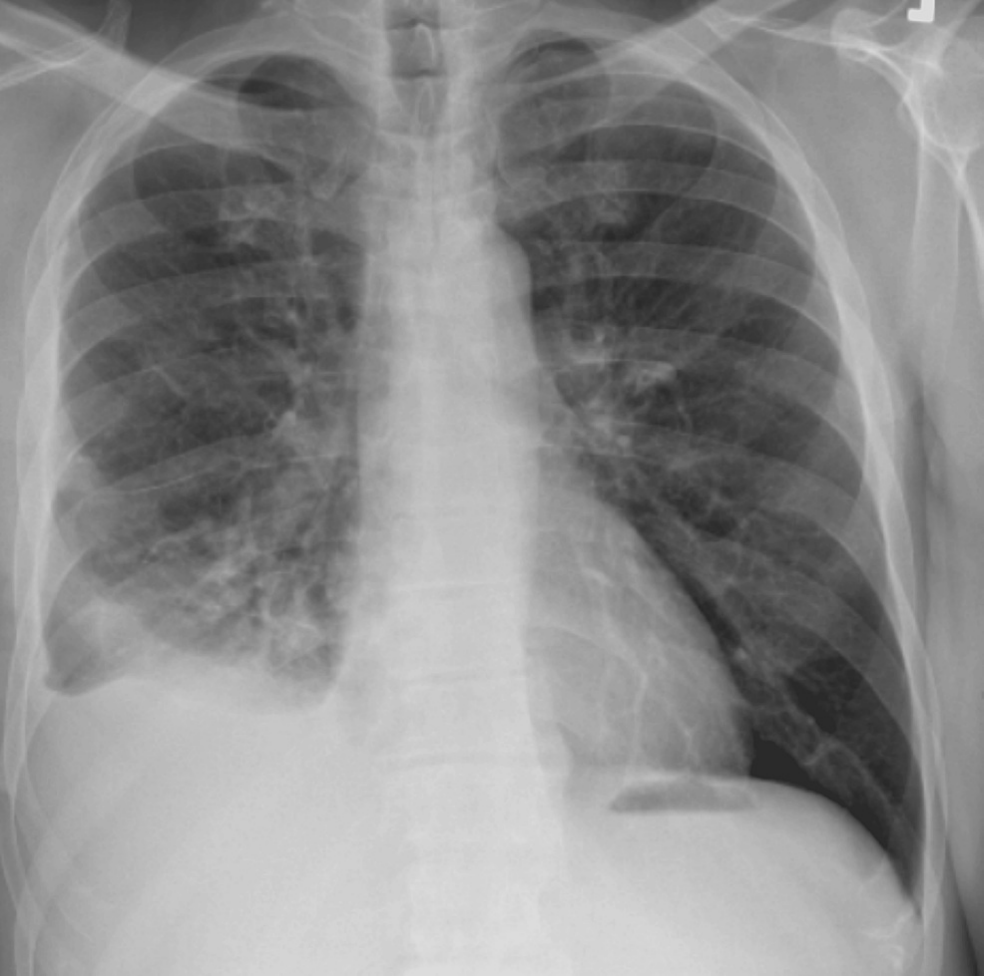
Chest X-ray showing significant interval improvement in aeration in the right lower lung parenchyma, mild right-sided pleural effusion still present tracking along the lateral wall.

He required vasopressor support for septic shock with the highest leukocytosis of 67 and received two days of albumin. He was extubated on POD 2, and his septic shock and pressure requirement improved. One of the right chest tubes was removed on POD 4, and the remaining on POD 5 without complications. He remained stable and was downgraded to the medical/surgical unit on 8/31/2022 and accepted for transfer to Medicine. His repeat testing for COVID-19 returned negative. He received an approximate total of 14 days of the new antibiotic regimen. Discharged on POD 13 on oral augmentin. He was seen in the clinic for three visits, showing significant improvement clinically and radiologically (Figure 4).

## Discussion

*P. micra* was initially identified and classified as *Peptostreptococcus micros*. The species was then re-named as *Micromonas micros* in 1999 and then again as *Parvimonas micra* in 2006. It is the only species in the *Parvimonas* genus [1,2].

It is a gram-positive coccus, an obligate anaerobe that resides mainly in the human oral cavity as a commensal pathogen, most abundant in the subgingival dental plaque [1,2].

Pulmonary infections caused by *P. micra* can range from abscesses and pleural effusions with progression to empyema. Poor dental hygiene has been identified as a risk factor. The main risk factors for infection may include dental procedures such as periodontitis, tooth extraction, or dental caries. In oral infections, the pathogenicity of *P. micra* has been attributed to their adhesion to cells, morphotype, and proteolytic activity. However, to date, the isolated infections, and these factors listed remain unclear [3].

The diagnosis of *P. micra* infection is mainly based on the culture of an adequate sample obtained from the site of infection. The culture of drainage or aspiration fluid, tissue samples, or blood cultures are adequate for the diagnosis of *P. micra*. Infections caused by *P. micra* are rare and require a high index of suspicion because of their non-specific symptoms and insidious evolution [3].

In a study by Hatta et al. demonstrated increased tumorigenesis properties caused by *P. micra* infection in the colon [1]. Previous studies discovered that *P. micra* is often sensitive to metronidazole, penicillin, amoxicillin-clavulanate, and clindamycin [4,5].

The evidence available for *P. micra* is limited to case reports, literature, and systematic reviews. Through our literature review, there were no cases reported with COVID-19 pneumonia and superinfection by *P. micra* causing lung abscess/empyema. Our patient was treated with cephalosporin and azithromycin initially. The American Association for Thoracic Surgery (AATS) recommends empiric treatment of community-acquired acute empyema with third-generation cephalosporins and metronidazole or IV penicillin with a beta-lactamase inhibitor. The duration of therapy for empyema has not been studied in comparative trials. The final duration should be determined by the organism's sensitivities, the adequacy of source control, and the response to therapy [6]. The usual initial management consists of placing a tube thoracostomy for drainage; large versus small caliber tubes did not show any difference in terms of mortality and prognosis. The goal of surgery is the removal of pus from the pleural cavity and achieving adequate lung expansion. In patients requiring surgical intervention, video-assisted thoracoscopy is the standard of care as long as there are no contraindications (such as the inability to tolerate single lung ventilation, inadequate source control/lung expansion via minimally invasive approach, and uncontrolled bleeding) [7].

## Conclusions

The pathogenesis of isolated *P. micra* infection and co-infection with COVID-19 remains unclear. Our case highlights a rare and interesting case of pleural empyema caused by a superimposed bacterial infection with *P. micra* in a patient with COVID-19 pneumonia, that was successfully managed after appropriate surgical intervention. Timely and accurate diagnosis, identification of causative pathogens, and appropriate management are critical in effectively treating pneumonia with empyema, thereby improving patient outcomes.
